# Uncovering the genetic diversity of *Giardia intestinalis* in isolates from outbreaks in New Zealand

**DOI:** 10.1186/s40249-022-00969-x

**Published:** 2022-05-04

**Authors:** Paul Ogbuigwe, Patrick J. Biggs, Juan Carlos Garcia-Ramirez, Matthew A. Knox, Anthony Pita, Niluka Velathanthiri, Nigel P. French, David T. S. Hayman

**Affiliations:** 1 School of Veterinary Science, Massey University, Palmerston North, Manawatu-Wanganui New Zealand; 2 School of Natural Sciences, Massey University, Palmerston North, Manawatu-Wanganui New Zealand

**Keywords:** DNA sequencing, Epidemiology, Genetic diversity, Giardiasis, Metabarcoding

## Abstract

**Background:**

*Giardia intestinalis* is one of the most common causes of diarrhoea worldwide. Molecular techniques have greatly improved our understanding of the taxonomy and epidemiology of this parasite. Co-infection with mixed (sub-) assemblages has been reported, however, Sanger sequencing is sometimes unable to identify shared subtypes between samples involved in the same epidemiologically linked event, due to samples showing multiple dominant subtypes within the same outbreak. Here, we aimed to use a metabarcoding approach to uncover the genetic diversity within samples from sporadic and outbreak cases of giardiasis to characterise the subtype diversity, and determine if there are common sequences shared by epidemiologically linked cases that are missed by Sanger sequencing.

**Methods:**

We built a database with 1109 unique glutamate dehydrogenase (*gdh*) locus sequences covering most of the assemblages of *G. intestinalis* and used *gdh* metabarcoding to analyse 16 samples from sporadic and outbreak cases of giardiasis that occurred in New Zealand between 2010 and 2018.

**Results:**

There is considerable diversity of subtypes of *G. intestinalis* present in each sample. The utilisation of metabarcoding enabled the identification of shared subtypes between samples from the same outbreak. Multiple variants were identified in 13 of 16 samples, with Assemblage B variants most common, and Assemblages E and A present in mixed infections.

**Conclusions:**

This study showed that *G. intestinalis* infections in humans are frequently mixed, with multiple subtypes present in each host. Shared sequences among epidemiologically linked cases not identified through Sanger sequencing were detected. Considering the variation in symptoms observed in cases of giardiasis, and the potential link between symptoms and (sub-) assemblages, the frequency of mixed infections could have implications for our understanding of host–pathogen interactions.

**Graphical Abstract:**

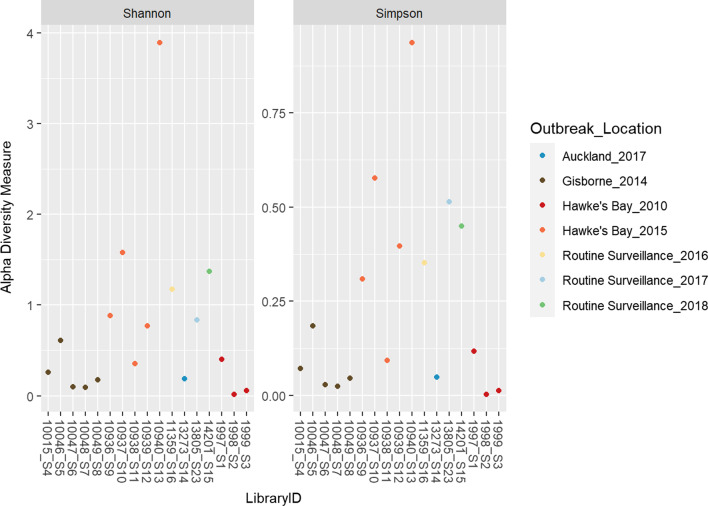

**Supplementary Information:**

The online version contains supplementary material available at 10.1186/s40249-022-00969-x.

## Background

*Giardia* is an enteric protozoan parasite with the distinction of being among the most common causes of diarrhoea in humans and farm animals worldwide [1]. Approximately 280 million people are reportedly infected with this parasite every year, and the prevalence of infections in humans ranges between 0.4 and 7.5% in high-income countries, and 8–30% in low and middle-income countries [2, 3].

*Giardia* infects the epithelial cells of the gastrointestinal tract. Infection usually results in self-limiting diarrhoea, however symptoms range in severity and infection in humans is also often asymptomatic [4, 5]. Infection can lead to chronic diarrhoea in immunocompromised individuals [1] and be detrimental to the growth and development of young children, particularly those in low resource settings where the possibility of infection in the first 2 years of life is relatively high [6, 7]. This is why giardiasis, the disease for which *Giardia* is the causative agent, was recognised by the World Health Organisation (WHO) in its neglected diseases initiative, highlighting the public health significance of this parasite [8]. Following that designation, reporting of this parasite has improved in many countries. Exacerbating the burden of this disease is the lack of any effective vaccines against the pathogen.

At present eight species of *Giardia* are recognised, these are: *G. agilis* (associated with amphibians), *G. ardeae* (great blue herons), *G. cricetidarum* (hamsters), *G. intestinalis* (alternatively named *G. duodenalis* or *G. lamblia*, discussed below), *G. microti* (associated with voles and muskrats), *G. muris* (rodents), *G. peramelis* (southern brown bandicoots), and *G. psittaci* (found in budgerigars) [9]. According to current understanding, the species responsible for all human infections is *G. intestinalis*, which is further divided into eight assemblages (or subtypes): A‒H. These assemblages can be further classified into sub-assemblages. Assemblages A and B are thought to be responsible for most zoonotic infections and cause the majority of infections in humans. However, as molecular techniques have advanced, evidence of infection by other assemblages has been identified in humans [2]. Assemblages A and B have a wide host range including humans, livestock, pets and wild animals. The remaining assemblages have narrow known host ranges. Assemblages C and D are associated with dogs and other canids, assemblage E with livestock, assemblages F with cats, assemblage G with rodents, and assemblage H with seals [2]. Assemblage B is responsible for the majority of human cases of giardiasis in low- and high-income settings, including in New Zealand where this assemblage was identified in 79% of cases between 2009 and 2015 [10].

Despite the variation in symptoms observed between individuals suffering from giardiasis, the mechanisms underlying these differences are poorly understood. Previous studies suggest that differences in infectivity exist between assemblages. Experimental observations found that human volunteers inoculated with assemblage B were more likely to succumb to infection and develop symptoms than those inoculated with assemblage A [11]. Nevertheless, studies looking at the correlation between symptoms and assemblages have produced contradictory results [12, 13]. The ability to link phenotypic features with assemblages would greatly increase our understanding of transmission patterns. Similarly, investigation of the genetic structure at the population level is essential for the proper inference of the transmission patterns and epidemiology of *G. intestinalis*.

*Giardia* is transmitted via the faecal-oral route and, in humans particularly, contact with contaminated water sources is the dominant mode of infection and cause of outbreaks [14]. Outbreaks of giardiasis occur frequently each year across the world. Previous reviews found that between 2011 and 2017 over 140 waterborne outbreaks of giardiasis occurred globally [15]. Outbreaks might be initiated through waterborne transmission but have the potential to spread further through human–human interaction [16]. Foodborne transmission is another dominant mode for the spread of *G. intestinalis.* Approximately 23.2 million cases of giardiasis attributed to contaminated food occur each year, and while few foodborne outbreaks have been recorded, this is thought to be due to limitations in detection and surveillance [9].

The true burden of this disease is potentially underestimated due to poor reporting in some countries. Giardiasis only became a notifiable disease in the USA, Europe and New Zealand between the late 1990s and early 2000s [17–19]. Increased surveillance will give a better idea of the true burden of giardiasis globally. Surveillance data in New Zealand found that *G. intestinalis* was responsible for 7.4% of total outbreaks in the country during 2016, with person-to-person contact being the most common mode of transmission [20]. There has, however, been an inability to identify the same subtypes of *G. intestinalis* in epidemiologically linked cases in New Zealand. A patient with an infection may carry multiple subtypes of the same infectious agent and the outcome of the competitive interactions between them has an effect on the clinical presentation of the disease, which, in turn, affects the efficacy of treatment [21]. For this reason, understanding the within-host genetic diversity of a pathogen is essential for effective disease management.

Questions remain as to whether epidemiologically linked cases in New Zealand were all part of the same events or if they represent within- and between-host diversity [10]. A possible reason for this could be a lack of resolution due to the standard detection methods used, such as enzyme immunoassays and Sanger sequencing. Because Sanger sequencing combines the contribution of all DNA fragments present in the reaction mixture, even this may lack sufficient resolution where mixed assemblages are present. PCR amplification of the *gdh* gene will amplify sequences from all *Giardia* subtypes present in the extracted DNA, which can lead to a mixed signal in the resulting Sanger sequence or failure to detect rare assemblage types if one is dominant. These limitations affect disease surveillance and make it difficult to capture within-host diversity. In contrast to Sanger sequencing, next-generation sequencing (NGS) techniques allow millions of fragments to be sequenced in a single run, granting the researcher the ability to separate the signal originating from each target molecule, leading to the efficient isolation, detection and quantification of rare types. In recent years, researchers have applied NGS techniques to study the epidemiology of giardiasis and other organisms, such as *Blastocytis*, which has led to great advances in the understanding of infectious diseases [22–24].

In this study, NGS metabarcoding techniques are used to gain a better understanding of the genetic diversity of giardiasis outbreaks in New Zealand. Taking faecal samples from three outbreaks of giardiasis that occurred between 2010 and 2018 in various regions across the country and some samples from routine surveillance, and utilising amplicon-based metabarcoding at the glutamate dehydrogenase (*gdh*) locus, the hypothesis that epidemiologically linked cases share subtypes undetectable with consensus sequencing technologies was tested. In addition, NGS was used to detect the degree of genetic diversity present in samples from patients diagnosed with giardiasis. Comparing these results to the results of Sanger sequencing at the same locus it was possible to detect the presence of mixed infections and gained a better understanding of the assemblages of *G. intestinalis* present in New Zealand. This study shows that amplicon-based sequencing provides better tools for painting a clearer picture of the role of genetic diversity in giardiasis outbreaks in New Zealand, which could lead to a better understanding of protozoan outbreak epidemiology.

## Materials and methods

### Sampling

The Protozoa Research Unit (PRU) at the Hopkirk Research Institute, Palmerston North, New Zealand, receives human faecal samples diagnosed positive by multiple accredited diagnostic laboratories from routine surveillance and outbreaks of giardiasis in New Zealand. All samples were anonymised before delivery to the PRU and were from patients diagnosed with giardiasis using enzyme immunoassays designed to detect the presence of *Giardia* antigens in faecal samples. For samples collected after 2015, diagnosis was done using multiplex PCR [25]. A list of the samples from routine surveillance and outbreaks of giardiasis that occurred in New Zealand between 2010 and 2018 and were delivered to the PRU can be found in Table 1.

**Table 1:** List of samples from outbreaks and routine surveillance along with the regions in which they occurred

Year	Region	Organism	Sample origin	Number of cases
2010	Hawke’s Bay	*Giardia*	Giardiasis outbreak	3
2014	Gisborne	Giardia*	Giardiasis outbreak	5
2015	Hawke’s Bay	*Giardia*	Giardiasis outbreak	5
2016	Christchurch	*Giardia*	Routine surveillance	1
2017	Auckland	Giardia*	Cryptosporidiosis outbreak	1
2017	Otago	*Giardia*	Routine surveillance	1
Total				16

Outbreaks where ‘Organism’ is annotated with (*) highlight situations in which *Cryptosporidium* and *Giardia* were identified in the same sample. A full list of the samples used in this study can be found in Additional file 1: Table S1

### DNA purification, end-point PCR and Sanger sequencing

Genomic DNA was extracted from faecal samples that had been stored at 4 °C using a Quick-DNA Faecal/Soil Microbe Kit (Zymo Research, Irvine, California, United States). The procedure required the use of a bead-beater (Tissue Lyser II, Qiagen) at 30 Hz for 5 min to disrupt the *Giardia* cysts. The purified DNA was stored at − 20 °C prior to further processing. A partial fragment of the glutamate dehydrogenase (*gdh*) gene was amplified by nested PCR using a previously established PCR programme and set of primers (Read, Monis and Thompson, 2004). Agarose gel electrophoresis was used to verify the presence of fragments of the correct size (432 bp) from all the PCR reactions. A blank containing deionised H_2_O was used as a negative control, and DNA from a sample that had already been verified by PCR and Sanger sequencing as containing *Giardia* DNA was used as a positive control. Sequencing of the amplification products was using Big Dye Terminator version 3.1 reagents and an ABI 3730XL automated DNA sequencer (Applied Biosystems, Foster City, California, USA) was used to characterise each sample at Massey Genome Services (Palmerston North, New Zealand).

### Metabarcoding next-generation sequencing (NGS)

The external *gdh* gene primers were modified to contain Illumina sequencing chemistry adapter sequences on the 5′ end according to standard protocols [26]. The PCR were run as above and products for all 16 samples were cleaned according to Illumina’s recommended protocols [26]. The DNA concentration in each sample was measured using a NanoDrop 2000 spectrophotometer (Thermo Fisher Scientific, Waltham, Massachusetts, United States), the samples diluted to a 5 ng/µl concentration for library preparation and amplicon-based sequencing. Sequencing was carried out on an Illumina MiSeq™ using 500-cycle V2 chemistry according to the manufacturer’s recommendations, producing paired reads of 250 base pairs. Due to the potential uneven representation of bases at each cycle with amplicon sequencing, an Illumina PhiX control library was loaded onto the Illumina MiSeq™ run at 20% volume, to even out the base composition and prevent biases in the initial few cycles that otherwise would result in base-calling errors.

### Construction of a *gdh* database

Using the *G. intestinalis* sequences from our in-house database a separate database was compiled consisting of 858 unique *gdh* sequences, most had previously been submitted to GenBank by our group [10] and can be found in GenBank with accession numbers MT265681–MT265802. The assembly of sequences and compilation of databases was done using Geneious (version10.2.6, Biomatters, Auckland, New Zealand) [27]. As the sequencing was carried out using the Sanger method, where there was a dominant peak it was used to assign assemblage, otherwise IUPAC DNA ambiguity codes used. To capture the greatest possible extent of the known diversity of *Giardia gdh* sequences, a dataset of all available *gdh* sequences for *G. intestinalis* from GenBank [28] was extracted and imported into Geneious. The search strategy employed one search string (*Giardia*) and included the keywords glutamate dehydrogenase, and *gdh*. The sequences were trimmed to the length of the primers employed in this study and all sequences less than 393 bp were discarded. This left 337 unique sequences from GenBank. The 337 GenBank sequences were combined with the 858 sequences extracted from our in-house database, then duplicate sequences were deleted to create a collection of 1109 unique sequences covering most of the assemblages of *G. intestinalis* that have been characterised at the *gdh* locus.

### Sequence processing

The Illumina sequence reads for the 16 samples involved in this study were analysed inside the Quantitative Insights Into Microbial Ecology 2 (QIIME 2) environment [29]. The dada2 methodology [30] was used to filter and trim the forward and reverse sequence reads, dereplicate them, calculate and plot error rates, merge paired reads and construct a sequence table, and remove chimeras. Then our database of 1109 known unique sequences was used as a reference to assign taxonomy to the merged sequences. To remove the impact of index hopping or PCR error, from the processed and merged sequences only the top 1971 sequences, based on the expected sequence length, were imported from dada2 into the phyloseq R package [31] for plotting, ranking of the most expressed sequences and creation of a heatmap. The resulting table of sequences was run against the reference database to exclude any sequences that did not match known sequences of *G. intestinalis*, then put through phyloseq again for further analysis. Only the top 50 sequences present across all the samples were used for the creation of bar plots and heatmaps to reduce the possibility of sequencing errors being included in the analysis.

## Results

### Overview of sample data

**Fig. 1:**
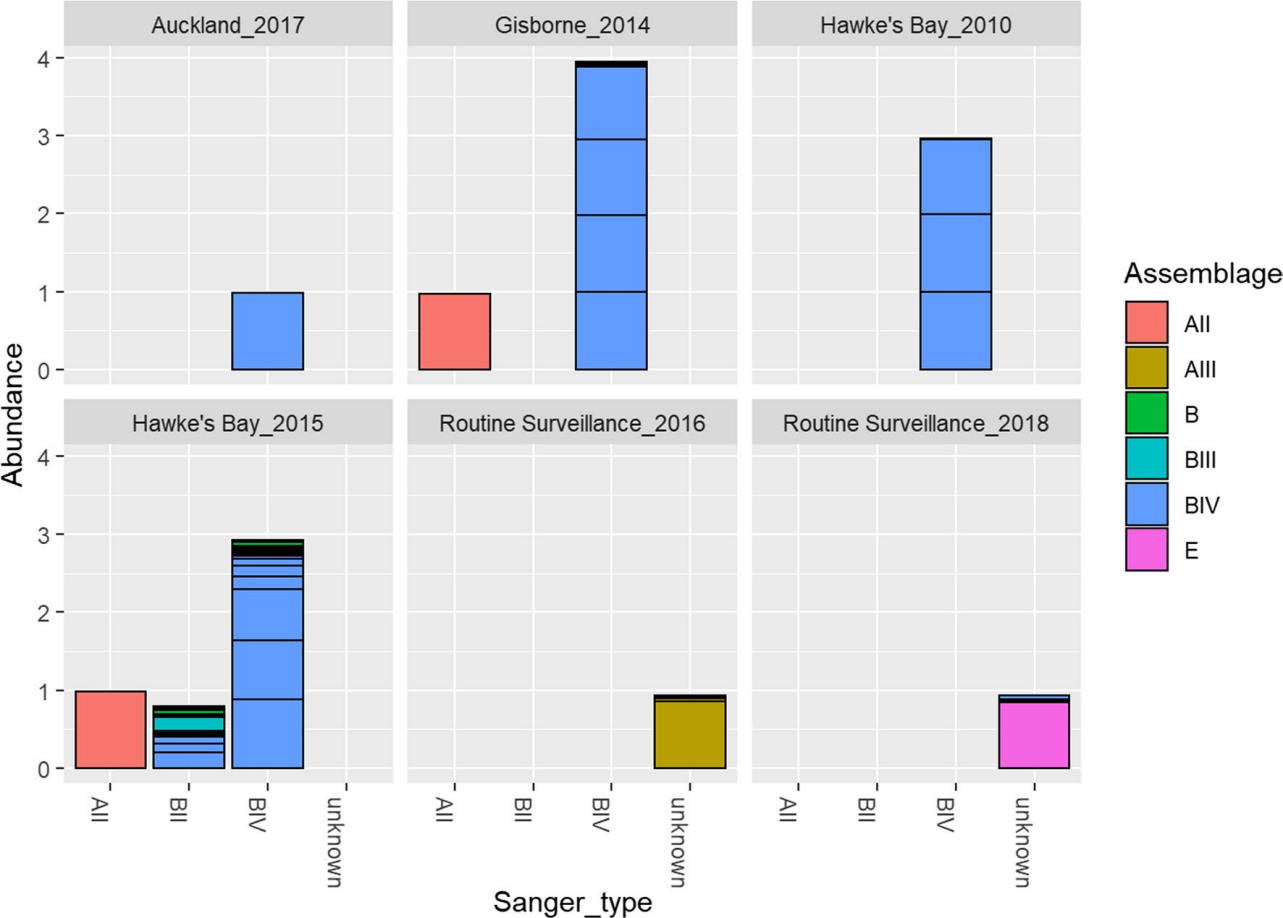
The taxonomic distribution of *Giardia intestinalis* (sub) assemblages in samples from the routine surveillance and the multiple outbreaks included in this study. The *x*-axis shows the (sub) assemblage of each sample according to Sanger sequence data and the *y*-axis displays the number of samples corresponding to each assemblage; the colour codes in each bar represent the genetic diversity within each sample according to NGS. *NGS* next-generation sequencing

Of the 16 historical faecal samples from cases of giardiasis that had occurred in New Zealand between 2010 and 2018, fragments of the *gdh* gene were successfully amplified for all of them using nested PCR. The samples for which the assemblage according to Sanger sequencing were known and the most dominant assemblage according to NGS are shown in Table 2. There were no disagreements in assigned dominant assemblage between the two sequencing methods. According to the NGS data, and focusing on the dominant assemblage in each sample, 11/16 samples were found to belong to sub-assemblage BIV, 1/16 to BIII, 2/16 to AII, 1/16 to AIII, 1/16 to E. Figure 1 provides a comparison of the assemblage assigned by Sanger sequencing and the diversity captured by NGS. It shows that even in genetically diverse samples, like the one from the outbreak in Hawke’s Bay in 2015, there are agreements between the Sanger sequence data and the NGS data. Analysis of the NGS data was conducted to probe the intra-sample diversity of these samples.

**Table 2:** Sample *Giardia* assemblages according to results of Sanger sequencing compared with most abundant assemblages according to NGS

Sample no.	ID	Sanger	NGS	NGS reads
1	1997	BIV	BIV	55,235 reads in 15,404 unique sequences
2	1998	BIV	BIV	113,042 reads in 21,061 unique sequences
3	1999	BIV	BIV	136,387 reads in 25,493 unique sequences
4	10,015	AII	AII	118,718 reads in 23,734 unique sequences
5	10,046	BIV	BIV	141,257 reads in 24,507 unique sequences
6	10,047	BIV	BIV	95,812 reads in 19,814 unique sequences
7	10,048	BIV	BIV	95,836 reads in 28,269 unique sequences
8	10,049	BIV	BIV	106,343 reads in 24,744 unique sequences
9	10,936	BIV	BIV	8184 reads in 3121 unique sequences
10	10,937	BIV	BIV	144,483 reads in 39,869 unique sequences
11	10,938	AII	AII	116,354 reads in 33,518 unique sequences
12	10,939	BIV	BIV	121,820 reads in 21,446 unique sequences
13	10,940	BIII	BIII	20,678 reads in 6331 unique sequences
14	11,359	Unspecified	AIII	112,267 reads in 19,785 unique sequences
15	13,273	BIV	BIV	103,784 reads in 22,832 unique sequences
16	14,201	Unspecified	E	75,624 reads in 15,918 unique sequences

“Unspecified” denotes samples for which the assemblage could not be determined. The number of reads generated by NGS from each sample after filtering, trimming and dereplication are shown for reference

*NGS* next-generation sequencing

### Metabarcoding analysis

**Fig. 2:**
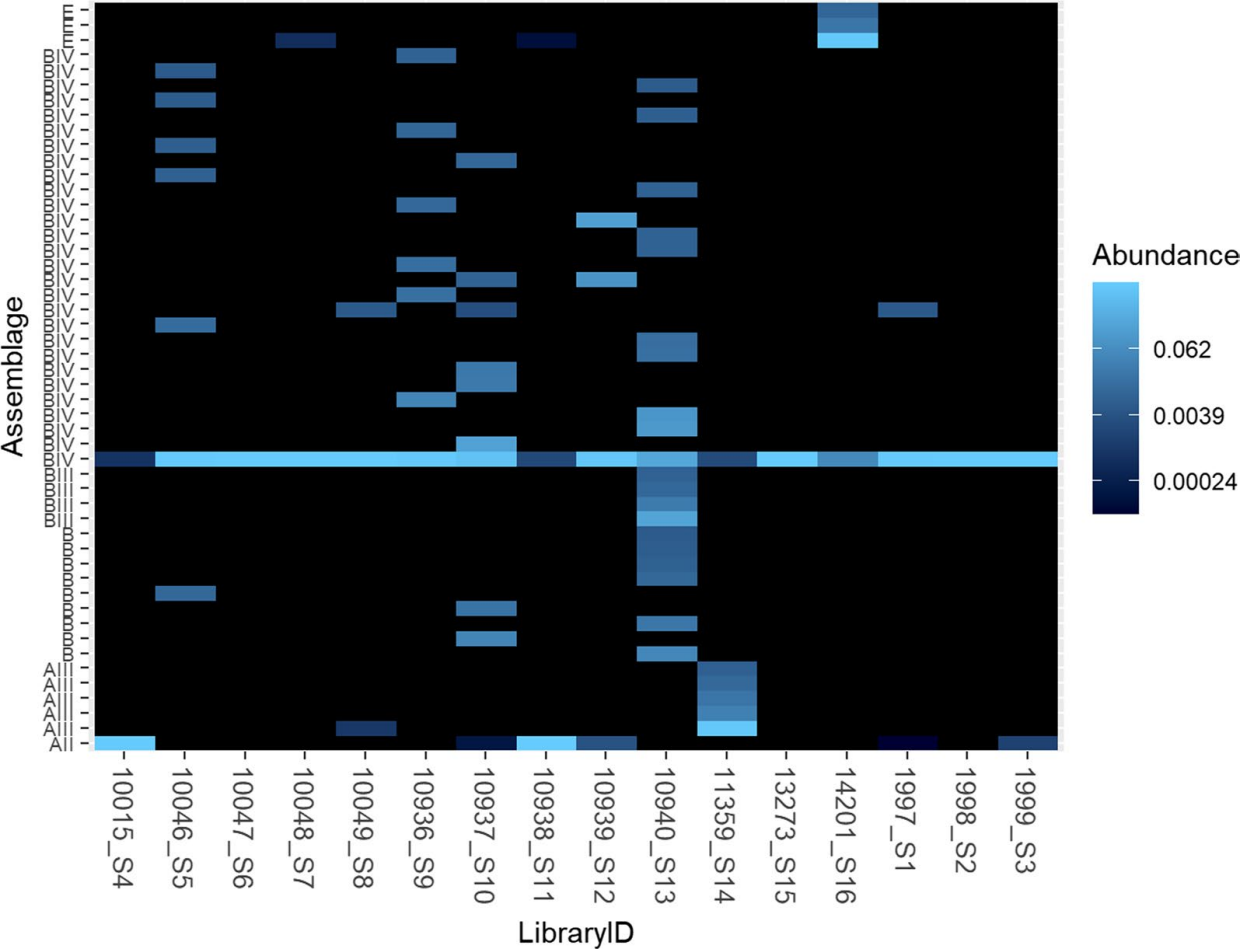
Heatmap showing the relative abundance of the top 50 *Giardia intestinalis* sequences in each sample. The multiple variants of each assemblage present in each sample are displayed on the *y*-axis. Each point on the *y*-axis corresponds to a unique sequence. This is why, in some cases, there are multiple sequences corresponding to one (sub) assemblage

The diversity of assemblages found in each sample after processing and analysis of the NGS reads are shown in Fig. 2. Similar to the results from the Sanger sequencing, the most abundant assemblage in most samples was assemblage B, specifically sub-assemblage BIV. This assemblage was present at some level in all samples. There was evidence of mixed infections in 13/16 samples. The majority of the genetic diversity within those 13 samples was due to the presence of multiple variants within assemblage B, for example, samples 10937_S10 and 10940_S13 showed evidence of multiple variants corresponding to assemblage B. The second most common assemblage present in this study was assemblage A, with 8/16 samples showing the presence of at least one variant of that assemblage.

Three samples from routine surveillance were included in this study (see Table 1) to compare the genetic diversity between samples from outbreaks and samples from sporadic cases. No appreciable differences were observed. Two samples (11359_S14 & 14201_S16) from routine surveillance represented the first report of sub-assemblage AIII and assemblage E in human samples from the South Island in New Zealand. These samples were analysed further in another study [32].

### Identifying links between outbreak cases

The primary aim of this study was to utilise NGS to detect a genetic link between epidemiologically linked giardiasis cases. To this end, an analysis of the outbreaks that occurred in Gisborne in 2014 and Hawke’s Bay in 2015 was conducted. These outbreaks were selected based on the fact that although the samples within each outbreak were epidemiologically linked, according to Sanger sequence data the samples did not share the same dominant genotype.

**Fig. 3:**
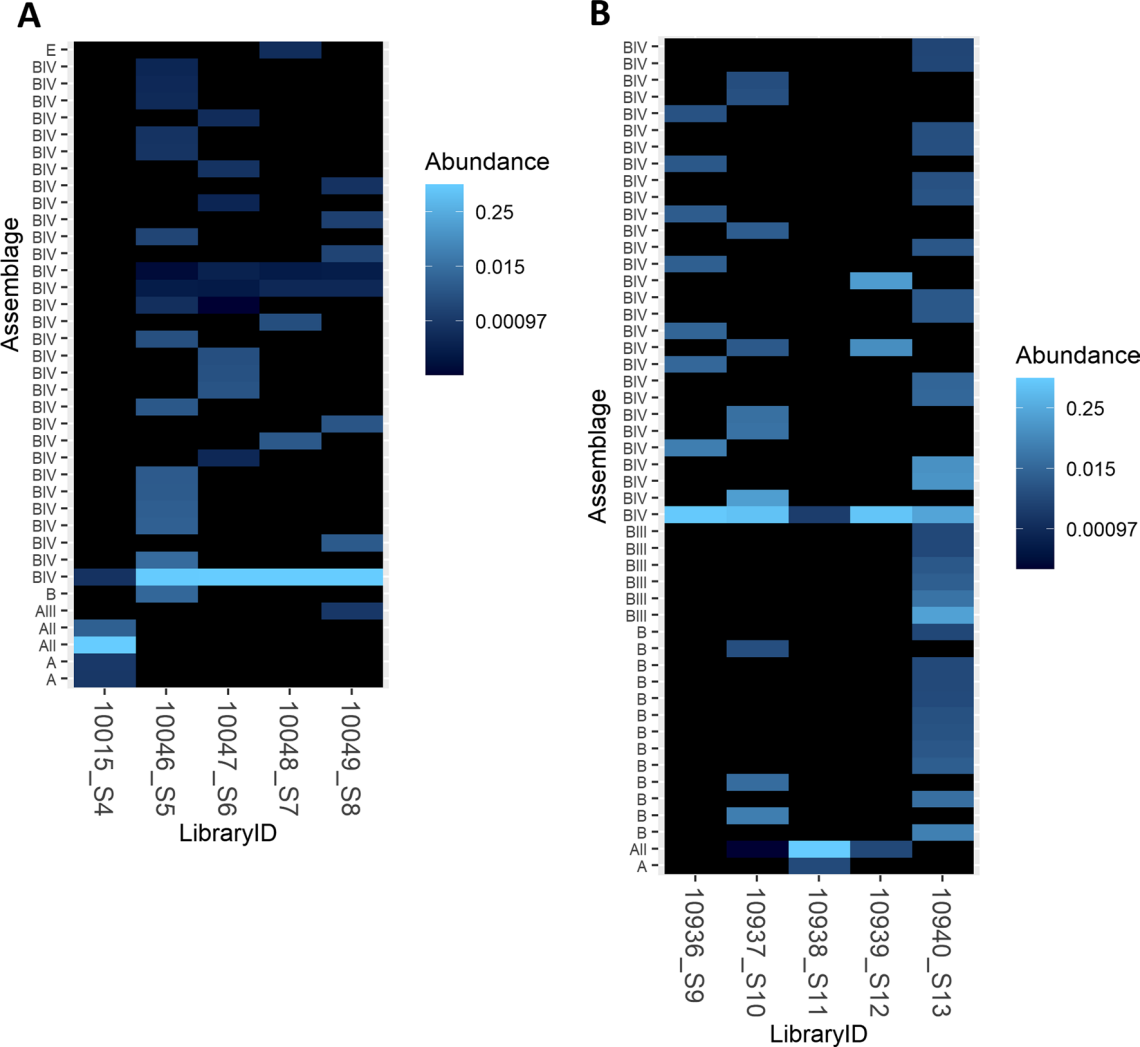
Heatmap showing the relative abundance of the top *G. intestinalis* sequences in each sample from the outbreaks of giardiasis that occurred in Gisborne in 2014 (**A**) and in Hawke’s Bay in 2015 (**B**). The multiple variants of each assemblage present in each sample are displayed on the *y*-axis. Each point on the *y*-axis corresponds to a unique sequence. This is why, in some cases, there are multiple sequences corresponding to one (sub) assemblage

Of the five samples from the outbreak that occurred in Gisborne in 2014, 4/5 were characterised as sub-assemblage BIV and 1/5 as AII according to Sanger and NGS data. Figure 3A is a heatmap showing the genetic diversity, captured by NGS, within the samples involved in this outbreak. From this, it is evident that a single variant of sub-assemblage BIV is shared by all the samples in this outbreak. Also, a copy of sub-assemblage AIII is present in one of the samples (10049_S8) and a copy of assemblage E is present in another of the samples (10048_S6) from this outbreak.

Of the five samples received from the outbreak in Hawke’s Bay in 2015, 3/5 were identified as sub-assemblage BIV, 1/5 as BIII, and 1/5 as AII according to Sanger and NGS data. From the heatmap shown in Fig. 3B it is evident that, despite the differences in dominant assemblages, sub-assemblage BIV is shared between all the samples from this outbreak. Sample 13273_S15 represented the only sample from an outbreak of cryptosporidiosis in Auckland. According to the NGS data, this sample was also positive for *G. intestinalis* sub-assemblage BIV (Fig. 2). This represents an example of a mixed-species infection. The NGS abundance data for the rest of the outbreaks is available in Additional file 2: Fig. S1.

## Discussion

This investigation into the intra-sample diversity of *G. intestinalis* in patients from historical outbreaks of giardiasis in New Zealand compares the capabilities of NGS and Sanger sequencing technologies. The strength of Sanger sequencing lies in its ability to detect the dominant sequence within a sample. The results outlined here show that NGS is also capable of the same level of discernment with regards to the identification of dominant sequences, shown by the agreements between the data from Sanger sequence and NGS of samples from cases of giardiasis that occurred in New Zealand between 2010 and 2018. The aim of this study was to use NGS to capture the diversity within samples, and this is where the benefit of NGS over Sanger shows itself. NGS is capable of sequencing multiple reads in each sample, compared to the one consensus read per sample achieved with consensus sequencing technologies. Here, NGS was employed to uncover the genetic diversity present within cases of giardiasis in this country. The clinical manifestation of giardiasis can differ between individuals. Further work needs to be done to ascertain if there is a link between (sub)assemblage and potential combinations of these and clinical presentation. This work may require samples from asymptomatic or mild cases as well as these with more severe clinical signs, however recent advances in the in vitro culture of *Giardia* [33] have the potential to help address this in vitro. So, the ability to capture the genetic diversity within samples from cases of giardiasis and link them to the symptoms displayed by the patient could greatly advance our understanding of the disease mechanisms of this parasite.

The data presented here suggest that during this time period assemblage B was the most common assemblage of *G. intestinalis* in New Zealand. However, the ability to capture the diversity of assemblages within samples showed that, although they might not be dominant, assemblages A and E reported with increased frequency in New Zealand, as evidenced by their presence in 7/16 and 4/16 samples respectively. This is particularly significant since assemblage E was thought to be exclusively infectious to livestock. However, recent studies have shown that it is increasingly present in humans as well [32, 34].

The subtyping in this study was carried out at only the *gdh* locus. This presents a potential limitation since other studies have shown that sequencing typing at different loci can result in the assignation of multiple subtypes [2, 35] and is why more studies are utilising multilocus sequence typing (MLST) [36]. However, this study sought to compared data from NGS to samples that had previously been characterised by Sanger sequencing at the *gdh* locus. For this reason, metabarcoding at the same locus was considered appropriate for this study. No-template controls or DNA extraction reagent blanks were not included in the library prep for NGS. These are usually used as an indication of the level of lane-hopping or environmental contamination present in the sequenced samples. Here, the use of nested PCR resulted in the amplification of specifically the *Giardia* DNA at the specific locus analysed in this study. In addition, while index hopping might be present it is usually between 0.1 and 1% on the Illumina MiSeq platform [37–39], NGS sequencing in this study produced millions of reads and the low quality and abundance reads were removed from the study. Furthermore, only the top 50 sequences were used when analysing the diversity across all samples and within each outbreak. Also, each outbreak had a different pattern of amplicons, generally with different dominant subtypes, which suggests there was little cross-contamination present. Another limitation was the low number of samples from outbreaks of giardiasis. This was because only a subset of samples from outbreaks that occurred in New Zealand between 2010 and 2018 are sent to our laboratory for molecular characterisation.

A key aim of this study was to use NGS to uncover genetic links between epidemiologically linked samples. It was hypothesised that epidemiologically linked cases share assemblages undetectable with consensus sequencing technologies. The outbreaks that occurred in Gisborne in 2014 and Hawke’s Bay in 2015 provided a perfect case study for this. In those outbreaks there were multiple dominant assemblages present in the samples within each outbreak. By applying NGS metabarcoding it was shown that sub-assemblage BIV was shared between all samples from the Gisborne and Hawke’s Bay outbreaks, thereby verifying the hypothesis. This improves our understanding of the epidemiology of these outbreaks.

These results show that the application of NGS can provide a better understanding of the epidemiology of giardiasis by linking outbreaks and identifying emerging subtypes. Co-infections with mixed (sub-)assemblages have previously been documented [2, 13, 32]. While cloning of PCR amplicons is one accepted method for detecting hidden genetic variation in a population, NGS is an alternative [13]. The samples used in this study were anonymised. Further work should include patient data to ascertain the contribution that travel, ethnicity, socioeconomic and other risk factors have on transmission patterns and epidemiological outcomes, particularly during outbreaks. For example, by analysing risk factors and *Giardia* (sub-)assemblages among patients within an outbreak it would be possible to ascertain if the incident was due to a subtype of *Giardia* common to another country, thereby allowing the assessment of the relative contribution travel makes to the disease within New Zealand [40]. Additionally, such analysis may show the relative contribution of zoonotic transmission by comparing the diversity and abundance of subtypes found in humans and livestock. As New Zealand has a substantial livestock industry, such an analysis would be able to aid public health bodies in developing strategies to mitigate the spread of giardiasis from animals to humans and vice versa.

## Conclusions

This study highlights the importance of utilising NGS technologies to uncover the genetic diversity of *G. intestinalis* in humans to gain a better understanding of the risk factors associated with the disease. Out of 17 samples, 13 showed the presence of multiple subtypes of *G. intestinalis*. This suggests that labelling a human sample using consensus sequencing technologies as belonging to one assemblage is insufficient and does not capture the true genetic diversity that can exist in one individual. In addition, these results suggest that *G. intestinalis* frequently invades humans as part of a mixed infection. This diversity has lead to mixed genetic signals, which do not reflect the epidemiological linkages when using Sanger sequencing, whereas NGS metabarcoding has allowed these to be made. This diversity may be clinically relevant, and requires further study. This will give us a better understanding of the disease mechanisms of the parasite and create a clearer epidemiological picture that will inform public health services in the development of better strategies to combat this persistent and prevalent parasite by allowing them to properly pinpoint all potential sources of infections and disrupt transmission pathways.

## Supplementary Information

**Additional file 1: Table S1.** Sample metadata.

**Additional file 2: Figure S1.** Heatmap showing the relative abundance of the top *G. intestinalis* sequences present in samples from the outbreak of giardiasis that occurred in Hawke’s Bay in 2010. The multiple variants of each assemblage present in each sample are displayed on the *y*-axis. Each point on the *y*-axis corresponds to a unique sequence. This is why, in some cases, there are multiple sequences corresponding to one (sub) assemblage.

## Data Availability

Sequence data is available in the Sequence Read Archive with the Accession Numbers PRJNA716067 and PRJNA785019.
