# Supplementary material for: Uncovering the genetic diversity of Giardia intestinalis in isolates from outbreaks in New Zealand
Source: Infect Dis Poverty. 2022 May 4;11:49. doi: 10.1186/s40249-022-00969-x (PMC9066983; doi:10.1186/s40249-022-00969-x)
Supplement: Supplementary file 1 — Additional file 1: Table S1. Sample metadata. [file 40249_2022_969_MOESM1_ESM.docx]

**Appendix**

**Additional Table S1.** Sample metadata.

| Massey ID | LibraryID | N | Outbreak | Location | Yr | Sanger_type | Source | Description | Outbreak_Location |
| --- | --- | --- | --- | --- | --- | --- | --- | --- | --- |
| 1997 | 1997_S1 | 1 | TRUE | Hawkes Bay | 2010 | BⅣ | Human | 1997_S1 | Hawke's Bay_2010 |
| 1998 | 1998_S2 | 2 | TRUE | Hawkes Bay | 2010 | BⅣ | Human | 1998_S2 | Hawke's Bay_2010 |
| 1999 | 1999_S3 | 3 | TRUE | Hawkes Bay | 2010 | BⅣ | Human | 1999_S3 | Hawke's Bay_2010 |
| 10015 | 10015_S4 | 4 | TRUE | Gisborne | 2014 | AII | Human | 10015_S4 | Gisborne_2014 |
| 10046 | 10046_S5 | 5 | TRUE | Gisborne | 2014 | BⅣ | Human | 10046_S5 | Gisborne_2014 |
| 10047 | 10047_S6 | 6 | TRUE | Gisborne | 2014 | BⅣ | Human | 10047_S6 | Gisborne_2014 |
| 10048 | 10048_S7 | 7 | TRUE | Gisborne | 2014 | BⅣ | Human | 10048_S7 | Gisborne_2014 |
| 10049 | 10049_S8 | 8 | TRUE | Gisborne | 2014 | BⅣ | Human | 10049_S8 | Gisborne_2014 |
| 10936 | 10936_S9 | 9 | TRUE | Hawkes Bay | 2015 | BⅣ | Human | 10936_S9 | Hawke's Bay_2015 |
| 10937 | 10937_S10 | 10 | TRUE | Hawkes Bay | 2015 | BⅣ | Human | 10937_S10 | Hawke's Bay_2015 |
| 10938 | 10938_S11 | 11 | TRUE | Hawkes Bay | 2015 | AII | Human | 10938_S11 | Hawke's Bay_2015 |
| 10939 | 10939_S12 | 12 | TRUE | Hawkes Bay | 2015 | BⅣ | Human | 10939_S12 | Hawke's Bay_2015 |
| 10940 | 10940_S13 | 13 | TRUE | Hawkes Bay | 2015 | BIII | Human | 10940_S13 | Hawke's Bay_2015 |
| 11359 | 11359_S14 | 14 | FALSE | Christchurch | 2016 | unknown | Human | 11359_S14 | Routine Surveillance_2016 |
| 13273 | 13273_S15 | 15 | TRUE | Auckland | 2017 | BⅣ | Human | 13273_S15 | Auckland_2017 |
| 14201 | 14201_S16 | 16 | FALSE | Otago | 2017 | unknown | Human | 14201_S16 | Routine Surveillance_2018 |

**Additional Fig. S1.** Heatmap showing the relative abundance of the top *G. intestinalis* sequences present in samples from the outbreak of giardiasis that occurred in Hawke’s Bay in 2010. The multiple variants of each assemblage present in each sample are displayed on the y-axis. Each point on the y-axis corresponds to a unique sequence. This is why, in some cases, there are multiple sequences corresponding to one (sub) assemblage.
