# Supplementary figures and images for: Uncovering the genetic diversity of Giardia intestinalis in isolates from outbreaks in New Zealand
Source: Infect Dis Poverty. 2022 May 4;11:49. doi: 10.1186/s40249-022-00969-x (PMC9066983; doi:10.1186/s40249-022-00969-x)

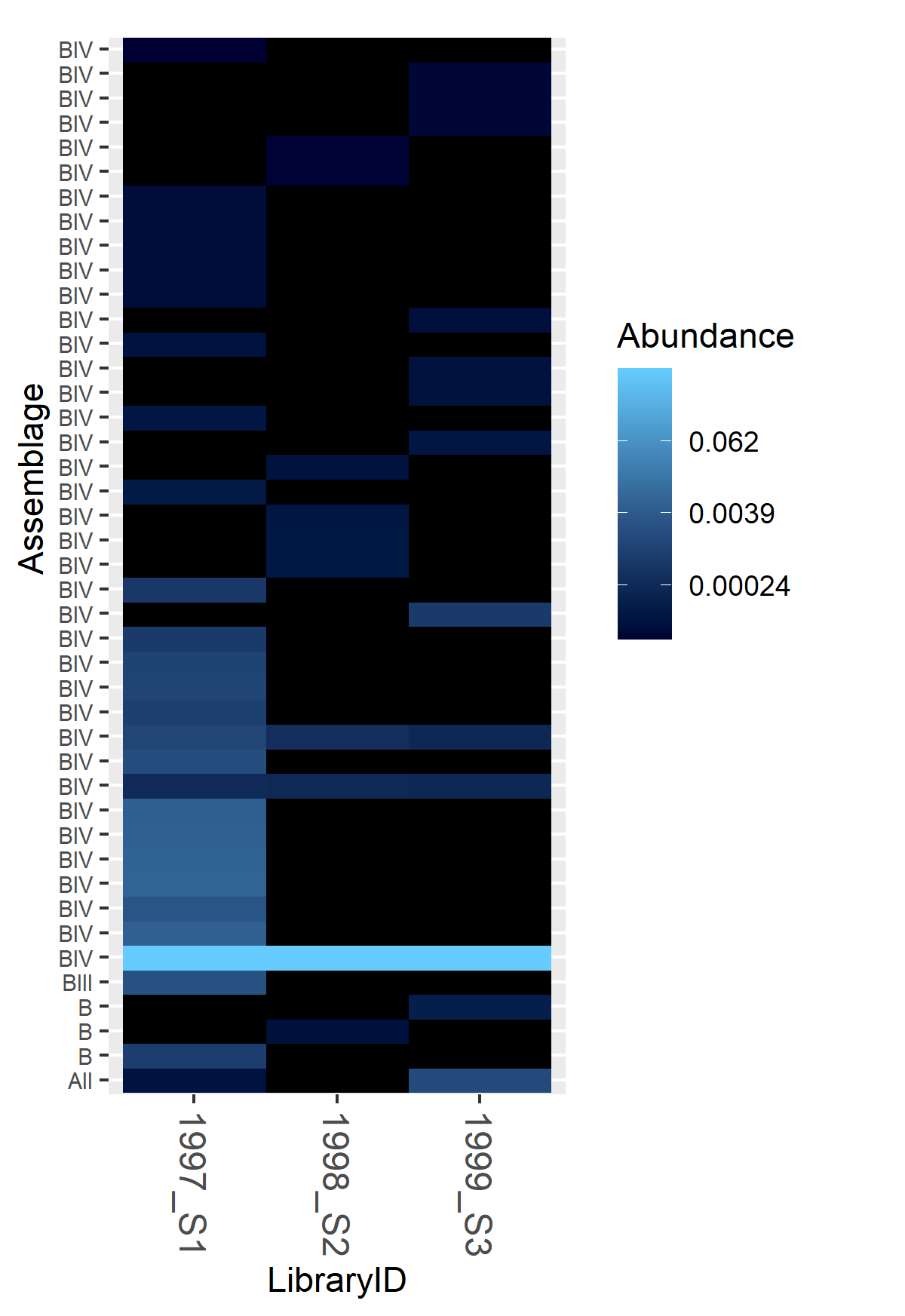

Supplement: Supplementary file 2 — Additional file 2: Figure S1. Heatmap showing the relative abundance of the top G. intestinalis sequences present in samples from the outbreak of giardiasis that occurred in Hawke’s Bay in 2010. The multiple variants of each assemblage present in each sample are displayed on the y-axis. Each point on the y-axis corresponds to a unique sequence. This is why, in some cases, there are multiple sequences corresponding to one (sub) assemblage. [file 40249_2022_969_MOESM2_ESM.png]
